# Photothermal effectiveness of microporous carbon nanospheres incorporated with polysulfone in direct contact membrane distillation†

**DOI:** 10.1039/d4ra05629a

**Published:** 2024-09-27

**Authors:** Moataz Morad, Mohamed S. Fahmi, Abdu Subaihi, Mohammed T. Alotaibi, Ahmed Shahat, Mohamed E. A. Ali

**Affiliations:** a Chemistry Department, Faculty of Sciences, Umm Al-Qura University Makkah 21955 Saudi Arabia; b Chemistry Department, Faculty of Science, Suez University P.O. Box: 43221 Suez Egypt; c Department of Chemistry, University College in Al-Qunfudhah, Umm Al-Qura University Saudi Arabia; d Department of Chemistry, Turabah University College, Taif University P.O. Box 11099 Taif 21944 Saudi Arabia; e Egypt Desalination Research Center of Excellence, Hydrogeochemistry Department, Desert Research Center Cairo 11753 Egypt m7983ali@drc.gov.eg

## Abstract

Carbon nano-spheres (CNS) were synthesized *via* a hydrothermal method using d-glucose as a precursor, followed by pyrolysis in a nitrogen atmosphere. The resulting CNS were integrated into polysulfone (PSF) membranes to enhance their photothermal properties. Characterization using various techniques revealed improved thermal properties upon CNS inclusion, with a notable increase in membrane surface temperature and enhancement of contact angle (CA) and liquid entry pressure (LEP). Composite PSF membranes containing varying CNS concentrations (0.25–5%) exhibited optimal performance at 3% CNS concentration, demonstrating enhanced morphological conformation and photothermal attributes. Evaluation under tungsten bulbs light using a photothermal membrane distillation system showed significant improvement in membrane distillation flux, achieving a maximum water flux of 7.73 L m^−2^ h^−1^ and a salt rejection rate of 99.9%. These findings highlight the potential of hydrothermal CNS in enhancing photothermal properties and membrane performance for applications in desalination and wastewater treatment.

## Abstract 2

Carbon nano-spheres (CNS) were synthesized *via* a hydrothermal method using d-glucose as a precursor, followed by pyrolysis in a nitrogen atmosphere.

## Graphical abstract

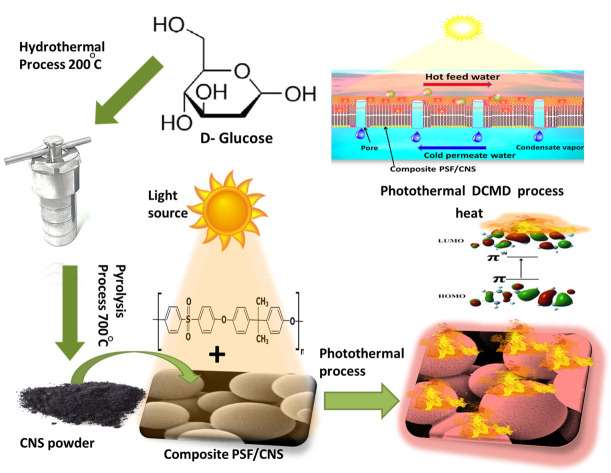

## 1. Introduction

**Fig. 1:**
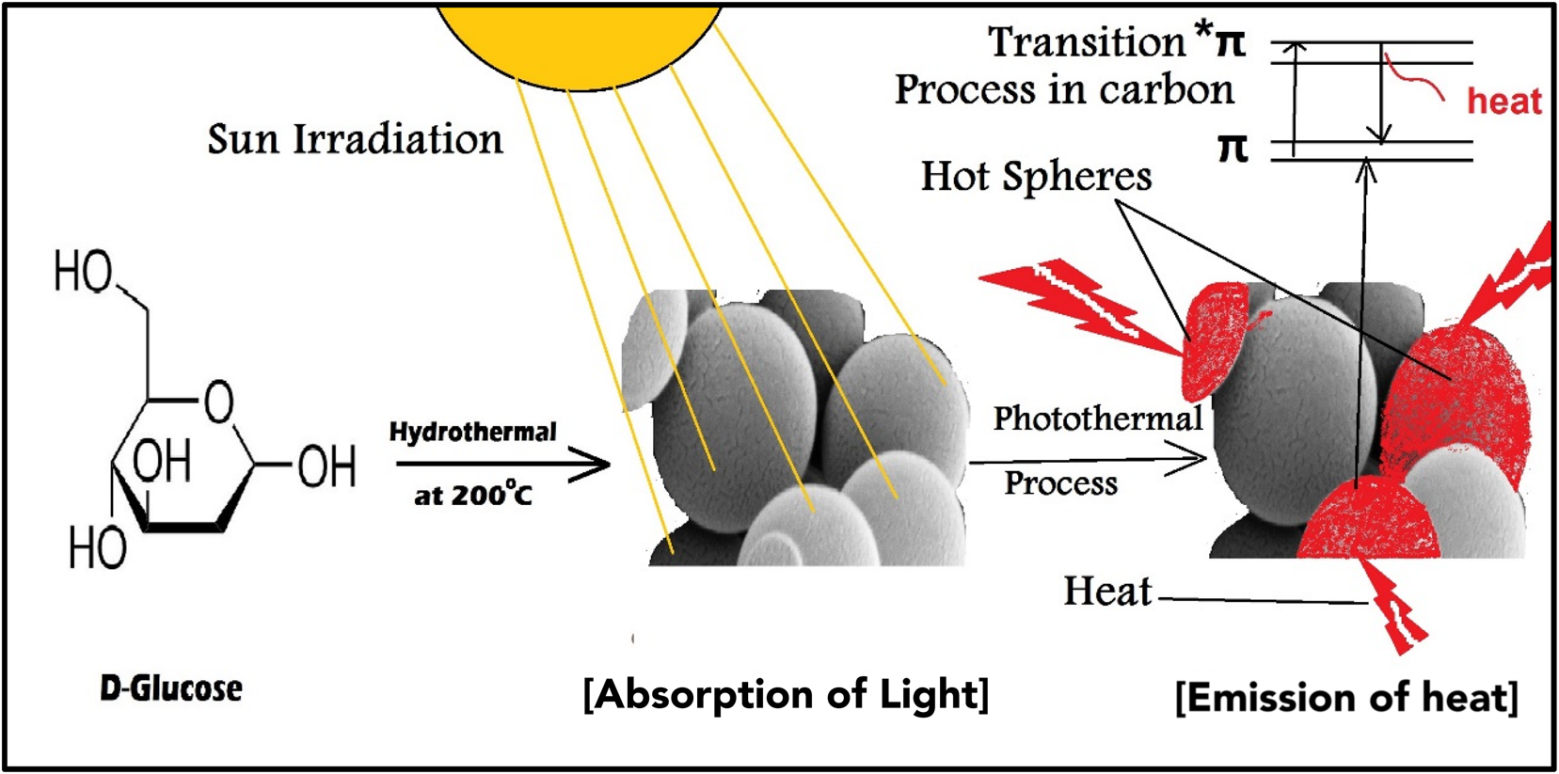
Schematic diagram depicts the synthesis and photothermal processes of CNS.

The use of nano-enabled photothermal absorbers has reignited interest in solar-powered water evaporation over the last five years. The cutting-edge photothermal materials have an exceptionally high photothermal conversion efficiency, allowing them to utilize the full spectrum of solar radiation.^1^ Photothermal materials efficiently absorb and convert light energy into heat, which can be used in multiple applications, such as phototherapy, photo imaging, and clean water production, and recently in membrane distillation applications.^2–4^ Photothermal material development has played a key role in solving water deficit challenges through water desalination in recent decades. The use of photothermal absorbers has revolutionized solar distillation, providing a modern and eco-friendly solution for purifying drinking water.^4,5^ While inorganic nanomaterials have an efficiency in light to heat conversion, they still have some problems. For example, the refractive index is less than two, *i.e.*, does not achieve a high light reflection (>11%) based on the Fresnel equation.^5^ On the other hand, thermoelectric materials such as carbon-enabled nano-absorber have both tightly held energy levels and loosely bound π electrons, enabling it to absorb the entire solar spectrum. The absorption of sunlight by the π electrons caused them to transition to the π* level, resulting in the release of heat energy as they returned to their ground state,^5,6^ as illustrated in Fig. 1. Various types of carbon-based absorbers, including graphene oxide (GO), carbon black, graphite, carbon composites, carbon nanotubes (CNTs), and amorphous carbons, have exceptional solar-to-heat conversion capabilities due to their unique inherent characteristics rather than metallic plasmon.^3,7^ In addition, carbon-based materials have been enabled as efficient vapor generators due to their exceptional photothermal ability, minimum reflection and emission, and excellent absorbance.^8^ Previous works have studied various types of photothermal materials, including metals, semiconductors, and carbon-based materials.^9,10^ By utilizing plasmonic resonance and other mechanisms, these engineered materials can convert incoming solar or photonic radiation into heat.^11^

The most common method for preparing the nanomaterials is the hydrothermal method, which can control the nanomaterials size and morphology.^12^ The morphology of the materials can be controlled by using either low-pressure or high-pressure conditions, depending on the vapor pressure of the main composition. The hydrothermal method allows for minimal material loss in the production of nanomaterials with high vapor pressures. Liquid phase or multiphase chemical reactions in hydrothermal synthesis allow for precise control over the compositions of synthesized nanomaterials. This special issue provides a platform for presenting the latest research outcomes on a hydrothermal synthesis of nanomaterials. Several papers on a hydrothermal synthesis of nanoparticles, nanorods, nanotubes, hollow nanospheres, and graphene nanosheets have been published in this special issue.^12^

Membrane distillation (MD) is a membrane process that operates based on thermal energy, where a heated aqueous solution is brought into contact with one side (known as the feed/retentate side) of a hydrophobic membrane.^13^ This membrane restricts the movement of liquid and forms a boundary between the vapor and liquid phases at the entry of the membrane pores. Volatile substances undergo evaporation, diffusion, and/or convection as they pass through the membrane, and subsequently condense on the opposite side of the membrane, known as the distillate or permeate. MD technologies provide significant benefits, including reduced operating temperatures, decreased operating pressure, high rejection rates, and performance that is not constrained by high osmotic pressure or concentration polarization. MD possesses features that make it highly appealing for both wastewater treatment and water desalination.^14^ Nevertheless, the conventional MD process is marked by significant disadvantages, such as the requirement for a substantial amount of energy to heat the bulk feed water, the loss of heat during the transportation of the feed from heating units to membrane modules, and the necessity of massive central pumping systems.^15^ Furthermore, the widespread use of this technology is hindered by its low thermal efficiency caused by temperature polarization (TP). This process is associated with the transmission of latent heat and conductive heat at the interface of the MD membrane.^16^ The temperature at the surface of the membrane is lower than the temperature of the bulk input water because of thermal conductivity and water evaporation.

In this study, we utilized the hydrothermal method to synthesize CNS. These CNS were then employed as photothermal nano-materials to absorb and convert photonic light into heat. This process aimed to enhance the heat transfer in both the surface and bulk of a polysulfone membrane. The goal was to overcome certain challenges associated with membrane distillation by employing a photothermal membrane distillation desalination (PMD) system. The PMD system usually employs a hydrophobic microporous membrane to separate the feed solution from the distillate, just like the conventional MD system. When exposed to sunlight, the photothermal nanoparticles in the PMD system generate heat at the evaporation surface. The temperature rise at the evaporation surface boosts the vapor pressure gradient across the membrane, driving vapor permeation. Condensation on the other side of the membrane collects the distillate after the vapor passes through. The photothermal process, one of the three conversion processes in the PMD system, converts light to heat. Hence, the overall performance of an efficient PMD system relies on both the solar-to-thermal conversion capability of photothermal nanomaterials and the intrinsic characteristics of the PMD configuration. Several strategies have been suggested to enhance the properties and efficiency of DCMD membranes.^17^

Through theoretical investigations, we will predict the performance of a photothermal evaporation system by considering factors such as salt accumulation, heat management simulation, and excellent structural design. The goal of this study is to help us understand how photothermal conversion works in various nano-absorbers. This will help the progress of solar-powered water evaporation and its uses in climate change, energy, and other areas.

The carbon nano-spheres (CNS) were utilized in the preparation of a photothermal PSF membrane distillation (PMD) system due to their exceptional ability to absorb light and efficiently convert it into heat due to black body proper and abundant of π-bonds between carbons atoms, thereby reducing heat loss and enhancing photothermal conversion efficiency. These materials outperform traditional nanomaterials by significantly improving solar-driven evaporation, addition to their porous structure, which allows for better light absorption and minimizes heat loss to the bulk water. Unlike other costly photothermal materials such as gold, silver, palladium, platinum, copper, and titanium, CNS offer an economical and more sustainable alternative, making them ideal for synthesizing polysulfone membranes with superior photothermal properties. This innovation contributes to the development of effective and cost-efficient membranes for clean water production using solar energy. Compared to previous research, this work represents a novel approach toward using more efficient and environmentally friendly materials to address the challenges of water and energy scarcity.^1^ In this work DCMD unit was modified as photothermal MD unit and all prepared composite membranes of polysulfone which doped with sonicated dispersed photothermal nanomaterials from CNS at different concentrations by phase inversion method. All prepared membranes were evaluated, characterized and investigated with different chemical techniques such as Raman spectroscopy, FT-IR, SEM, TEM, dynamic mechanical analysis (DMA), contact angle, UV-spectroscopy and IR-camera.

## 2. Material and methods

### 2.1 Materials

d-Glucose (Dextrose Anhydrous) analytical grade was purchased from Loba Chemie. Co. (India), polysulfone (PSF, Udel-3500) was supplied by Solvay Co. (Belgium), *N*,*N*-dimethylformamide (DMF) was brought by Fisher Chemical Co. (Pittsburgh, Pennsylvania), ethanol and NaCl were supplied by Adwic Co. (Egypt). Glass plate [25 × 30 cm] with thickness 0.3 cm was supplied by Saint-Gobain Co. (Egypt) and bar of steel as casting knife its groove depth 150 μm local manufacturing. All details of synthesis and instrumental characterization of CNS were documented in the ESI.†

### 2.2 Preparation of PSF and PSF/CNS composite membranes

The phase inversion method was used to prepare all the membranes.^18^ In brief; 15 g of PSF dried crystals were dissolved in 85 mL of DMF solvent (15% wt/v) and stirred for 6 hours at 100 °C. After getting a homogeneous solution, it was left for at least 6 hours before casting to ensure degassing. To prepare a composite membrane of polysulfone/carbon nanosphere (PSF/CNS), stock pure polysulfone solution was prepared then different concentrations of CNS (0.25%, 0.5%, 1%, 3%, and 5%) were sonicated in five separated conical for 1 h by Daihan sonicator at max level, after good dispersion PSF stock solution was divided into five conical equally. Nanomatrex of PSF/CNS is stirring for 15 minutes before casting. To carry out the phase inversion process, the casting solutions of PSF and PSF/CNS were cast onto a dry clean glass substrate at room temperature using a stainless-steel knife at a specific wet thickness of 150 μm. After that, the substrate with the nascent membranes was immersed in a coagulation bath of DI water at room temperature. The resulting membranes were removed from the water bath, washed several times with DI water and then kept dry for 24 h before use.

### 2.3 Evaluation of different PMD membrane performance

PMD unit compartments illustrated in (ESI, Fig. S1†), a high transparent acrylic (reflection index less than 3% was measured by using Mini Light Meter UT383) with a thickness of 2 mL was used to install the MD cell. The main components of the MD cell are; (i) feed and permeate tanks with a capacity of 1 L (ii) two peristaltic pumps with a max flow rate of 6 L min^−1^, (iii) thermocouples for monitoring the temperature on the membrane surface, (iv) flow meters to measure the flow rate of feed current, where the face of the module in this circuit is facing to the light source (a light bulb or the sun). All membranes were evaluated by PMD unit at constant operation conditions feed flow rate (FFR) (1.4 L min^−1^), feed temperature (*T*_f_) ranged from (35 °C to 55 °C), permeate temperature (*T*_p_ = 20 ± 3 °C), saline feed water salinity (FC) (2.0–40 g L^−1^) addition to Suez gulf water and various factors effects were investigated to obtain the best results and modifications.

A tungsten bulb with different electrical power capacities of 100 watts equivalent light intensity (LI) about 1650 Lumen ≈ 13.8 w m^−2^, 200 watts (LI = 3200 Lumen), and 300 watts (LI = 4500 Lumen) was used as a light source and fixed at a distance of 12 cm from the transparent surface of the membrane cell. The photonic intensity was calculated according to the square inverse law in eqn (1) about 958 w m^−2^ ≈ 1 sun for 100 watts bulb at 12 cm distance besides the sunlight spectrum.^19^1
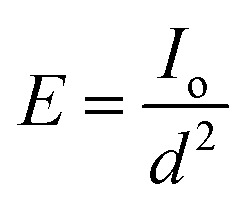
where *E* (w m^−2^) the light efficiency and *I*_o_ (w m^−2^) is light source intensity and *d* is light source distance (m). While the permeate flux, *J*_w_ (L m^−2^ h^−1^), was taken as the total volume of water flux obtained using the following eqn (2).2
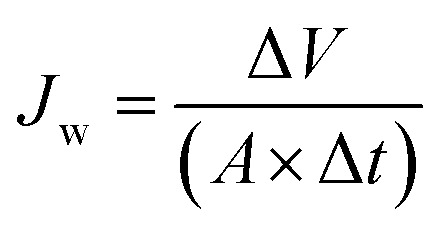
In the equation, Δ*V* represents the change in permeate volume (L), *A* is the membrane active area (0.00866 m^2^), and Δ*t* is the time of the experiment for collected flux (h). The salt rejection (*R*_s_ (%)) was determined using the following eqn (3):3
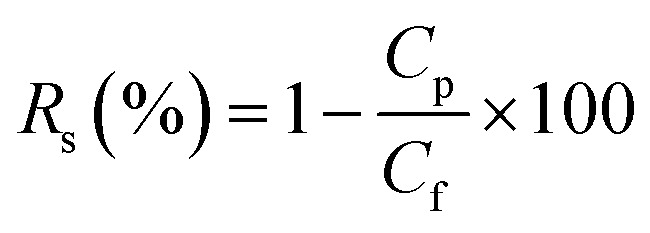
where *C*_p_ and *C*_f_ are the salts concentration of the permeate and feed solutions (mg L^−1^), respectively.^20,21^

Durability of the membranes: durability of this membrane was evaluated under certain operation condition where *T*_f_ ranged 50 ± 3 °C, *T*_p_ about 20 ± 3 °C, feed water tank concentration is about 4.0 g L^−1^ and tap water was used as permeate cold water with TDS about 340 mg L^−1^ all these operation conditions remained constant throughout the experiment in presence of light source with electrical power ability 100 watts at distance 12 cm (≈1 Sun). During the process of PMD, a saltwater sample with a salinity of 38 g L^−1^ was acquired from the Suez gulf. This sample was employed as a feed solution without undergoing any pretreatment. Add to that porosity (*ε*) of different prepared membranes was calculated and evaluated and documented in the ESI.†

## 3. Results and discussion

### 3.1 Characterization of carbon nano-spheres

**Fig. 2:**
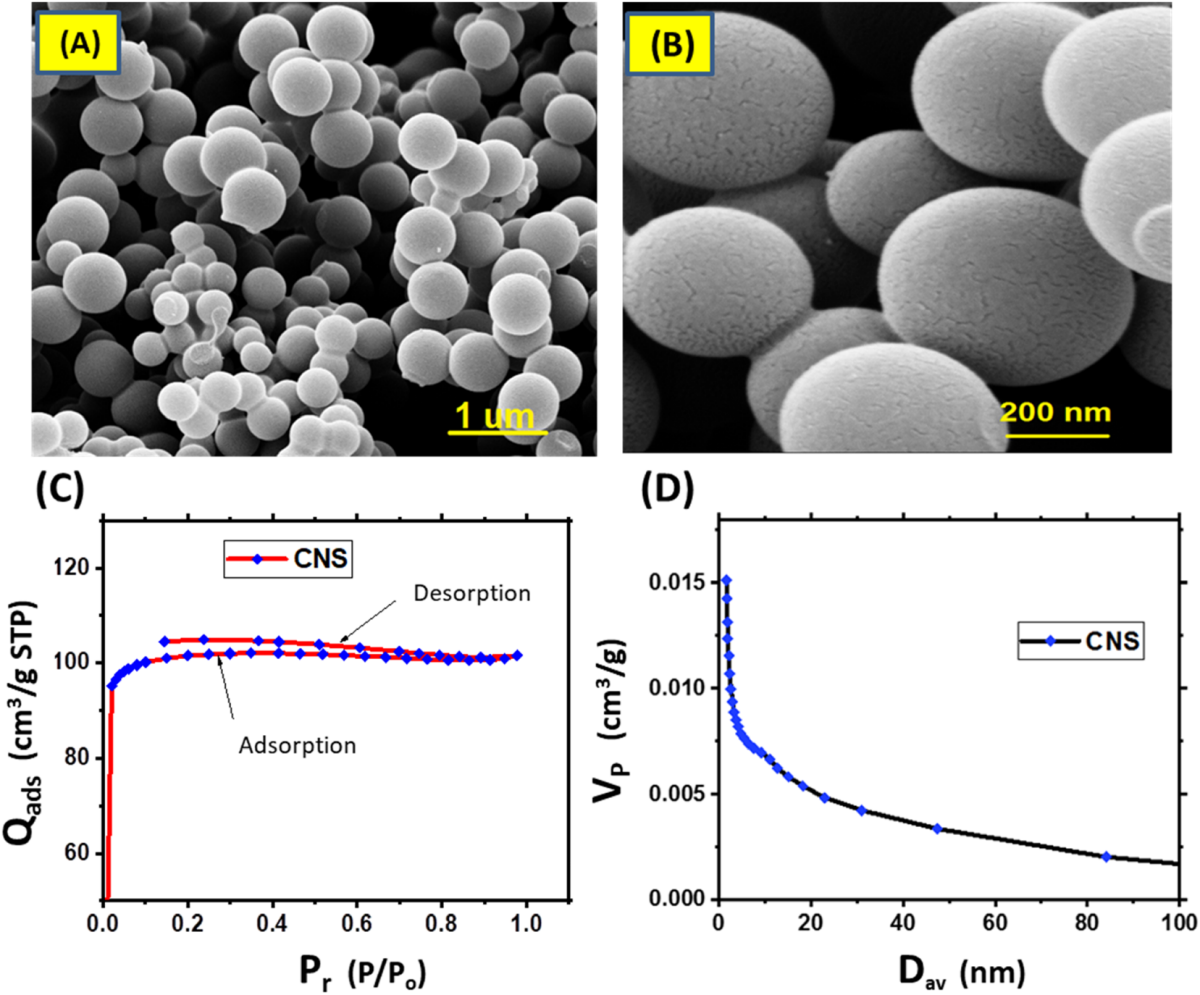
SEM images of CNS particles at different magnifications (A and B), isotherm adsorption of CNS sample (C) and BJH pore distribution of CNS (D).

The morphology and the nature porosity of the synthesized CNS that obtained from d-glucose and underwent to pyrolysis at a nitrogen atmosphere (to avoid oxidation process and formation active function groups) were emphasized using SEM and BET as illustrated in Fig. 2. As demonstrated in Fig. 2a and b, the SEM images show that CNS particles have a smooth surface clearly 3D porous spherical shape.^22,23^ This spherical and surface porosity of CNS particles could support photothermal process as light will penetrate for most C=C, added to that improve MD membrane morphology through forming passages, narrow voids for water vapor, and increase surface roughness, and decrease from surface tension energy. Thus, to obtain hydrophobic membranes.^24^Fig. 2c and d elucidates the Barrett–Joyner–Halenda (BJH) pore distribution and isotherm adsorption curve of CNS sample. It can be observed that the adsorption isotherm from type I which is given by microporous solids (CNS) and its pore size is not very much greater than the molecular diameter of the sorbate molecule according to IUPAC classification. Pore size distribution curve (BJH) exhibited a uniform mono-model distribution of pore size in the microporous.^22^Table 1 presented the analysis of the pores and BET surface area of CNS for multi-point samples. The results showed that the average surface area about 320.682 m^2^ g^−1^ Additionally, the pore volume (*V*_P_) and half of average pore diameter (*D*_av_) (radius) confirmed the isotherm curve findings, indicating that the spheres exhibited micropores in a range larger than the standard mesoporous range (20–500 Å).

**Table 1:** BET analysis and surface area of CNS materials

Sample	*S* BET (m^2^ g^−1^)	Pore volume (cm^3^ g^−1^)	Pore radius (nm)
Multi points	320.682	0.112	1.674

**Fig. 3:**
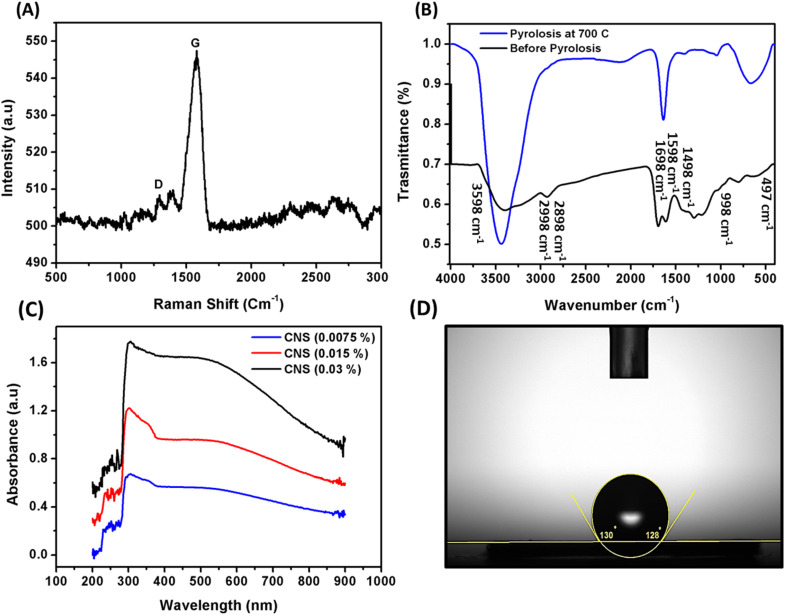
Raman shift spectra (A), FT-IR before and after calcination process (B), UV-spectroscopy of different concentrations (C), and contact angle (D) of CNS.

The application of the Raman spectrum confirmed the nature and chemical structure of the synthesized CNS particles. Fig. 3a shows that the CNS particles exhibited D and G bands at 1349 and 1589 cm^−1^. These bands correspond to sp^3^ carbon and sp^2^ carbon, respectively. This confirms the presence of C=C bonds in the carbon structure of the particles. Moreover, several peaks appear in the spectrum. A peak at 1470 cm^−1^ is due to sp^2^ vibrations, while a peak at 1170 cm^−1^ results from the stretching and isolation of the C–H bond. A peak at 2950 cm^−1^ arises from the combination of the D and G peaks. The peaks at 2740 and 2500 cm^−1^ are due to the overtones of the D peaks. Raman spectroscopy can extract structural and optical information of carbonaceous materials. The *I*_D_/*I*_G_ ratio correlates with the in-plane length of polyaromatic clusters (La), from TEM or X-ray diffraction, and relates to disorder in sp^2^ structures. The Tuinstra–Koenig relation is a key method for relating *I*_D_/*I*_G_ to La.^25^Fig. 3b elucidates the FT-IR spectrum of CNS before and after pyrolysis. The carbon spheres, before pyrolysis, showed a broad absorption band in the spectrum ranging from 2998 cm^−1^ to 3598 cm^−1^. This band corresponds to the stretching vibrations of O–H group. Additionally, a small band at 2898 cm^−1^ was observed, which corresponds to the stretching vibrations of C–H group. The vibrations of C=O and C=C are represented by the bands at 1598 cm^−1^ and 1698 cm^−1^, respectively. The bands between 998 and 1498 cm^−1^ indicate C–OH stretching and O–H bending vibrations, while the bands between 998 and 497 cm^−1^ signify C–H bending vibrations. The importance of HMF, LA, and other chemicals as intermediate products in the hydrothermal reaction of glucose was revealed by these bands.^26,27^ The results showed that aromatization and polymerization processes occurred during hydrothermal treatment of d-glucose, consistent with studies by Sevilla and Fuertes^28^ and Li *et al.*^29^ The FT-IR analysis of the carbon spheres grown after pyrolysis and subsequent calcination at 700 °C exhibited bands at 2898 cm^−1^, 1698 cm^−1^, and the 1998–1598 cm^−1^ region disappeared. This disappearance indicates the removal of most oxygen-containing groups from the spheres.^22^

Fig. 3c illustrates the behavior of CNS light band absorbance in the wavelength range about 200–900 nm, which showed broad curves bands at different concentrations from CNS. This confirms that CNS particles are effective as photothermal conversion agents at different wavelengths of light. They possess unique features such as tightly held energy levels and loosely held π electrons, which enable them to absorb the entire spectrum of light. The absorption of photonic light by these π electrons leads to a shift to the π* transition level, and subsequent release of energy as heat when returning to their ground state. The water contact angle is a commonly used measurement to determine the hydrophilicity or hydrophobicity of a material's surface. In this study, we used it to investigate the wetting properties of the synthesized CNS. The water contact angle of CNS was found to be 129°, as shown in Fig. 3d. This confirms that the CNS particles are hydrophobic, which can be attributed to their spherical shape and removal of most active functional groups during pyrolysis step, as observed in the FT-IR analysis.^6,18^

### 3.2 Characterization of PSF and PSF/CNS composite membranes

**Fig. 4:**
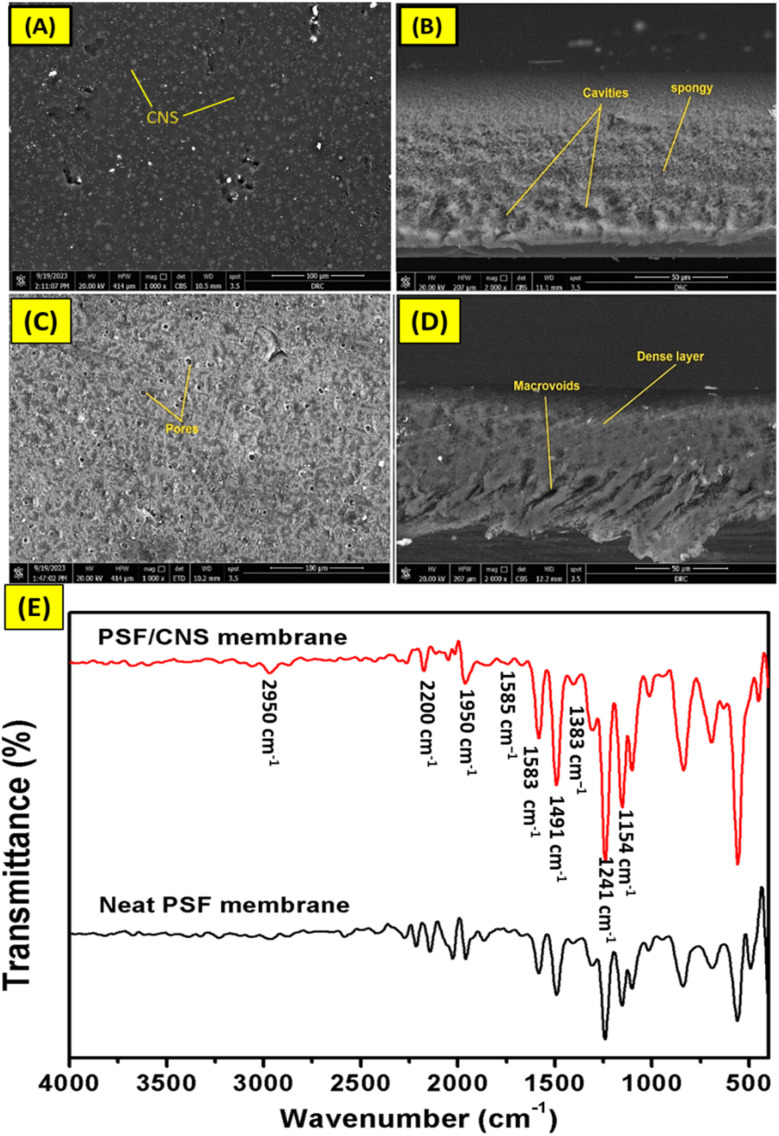
Surface SEM images of composite PSF/CNS and neat PSF membrane (A and C) and their cross-section (B and D), respectively and FT-IR spectra of composite PSF/CNS (3%) and neat PSF membrane (E).

Fig. 4a–d shows the surface and cross-sectional images of PSF and nanocomposite PSF/CNS membranes. The SEM images in Fig. 4a and b show the appearance of CNS on the surface, which largely works to create new voids for transportation of vapor molecules in the membrane. Also, its site, near or on the surface, assisted them in harvesting light spectrum with high efficiency. The change in the morphology of the membrane surface after adding CNS compared to the surface of the pristine PSF membrane is shown in (Fig. 4a and c). This can be interpreted as an enhancement in surface characteristics such as porosity, roughness, surface area, and hydrophobicity of the new composite membranes, which was confirmed by the increase in LEPs as shown in (ESI, Fig. S2†). Additionally, new photothermal surfaces capable of converting light spectrum to heat through CNS-doped particles on the surface were created.^1,18^ On the other side, the cross-section images (Fig. 4b and d) show the effect of nano modification on changing the morphological composition of the membrane before and after adding CNS particles. This is clearly appeared through the formation of cavities like spongy and the formation of gaps and decreases macro voids in neat membranes as a filler nanomaterial.^30^ These hydrophobic CNS materials hinder the pace at which nonsolvent and solvent exchange occurs, hence reducing water diffusion during the coagulation process. This allows for the formation of a more compact and porous structure, which acts as a pathway for vapor molecules and reduces tortuosity.^19^

Fig. 4e shows the FT-IR of PSF and PSF/CNS (3%) composite membranes, where the PSF/CNS membrane exhibited a slight difference compared to the neat PSF membrane. This is because of the overlapping of C=C bands of CNS and polysulfone chain. However, the FT-IR spectra shows all the characteristics bands of PSF such as di-aryl ether (Ar–O–Ar), and di-aryl sulfone (Ar–SO_2_–Ar), which appeared at 1154.37 cm^−1^ and 1241.88 cm^−1^, respectively. The bands at 1491.6 cm^−1^ and 1583.7 cm^−1^ belong to the vibration of the aromatic (C=C) in PSF molecule.^31,32^ The skeletal vibrations due to the conjugation can be seen at the bands of 1585 cm^−1^, 1383 cm^−1^, and 1148 cm^−1^ due to the asymmetric and symmetric stretching vibrations of the SO_2_ group. It is worth to mention that appearing of a small band at 2950 cm^−1^ for PSF/CNS membrane may be due to O–H group from humidity water, also its noticeable disappearing of some bands as at 1950 cm^−1^ to 2200 cm^−1^ and 2250 cm^−1^ as an impact of doped CNS overlapping vibrations bands with PSF bands. It's worth mentioning that the similarity in the FT-IR spectra of polysulfone membranes with and without CNS can be attributed to several factors. Firstly, the intrinsic properties of polysulfone, including its sulfone and aromatic groups, dominate the spectra. Secondly, carbon nanospheres were added in small amounts, their impact may be minimal. Thirdly, good dispersion of the CNS within the polysulfone matrix can result in negligible changes to the chemical bonds. Finally, the nanospheres may possess similar functional groups to those in polysulfone (like C=C), leading to overlapping spectral features.

**Fig. 5:**
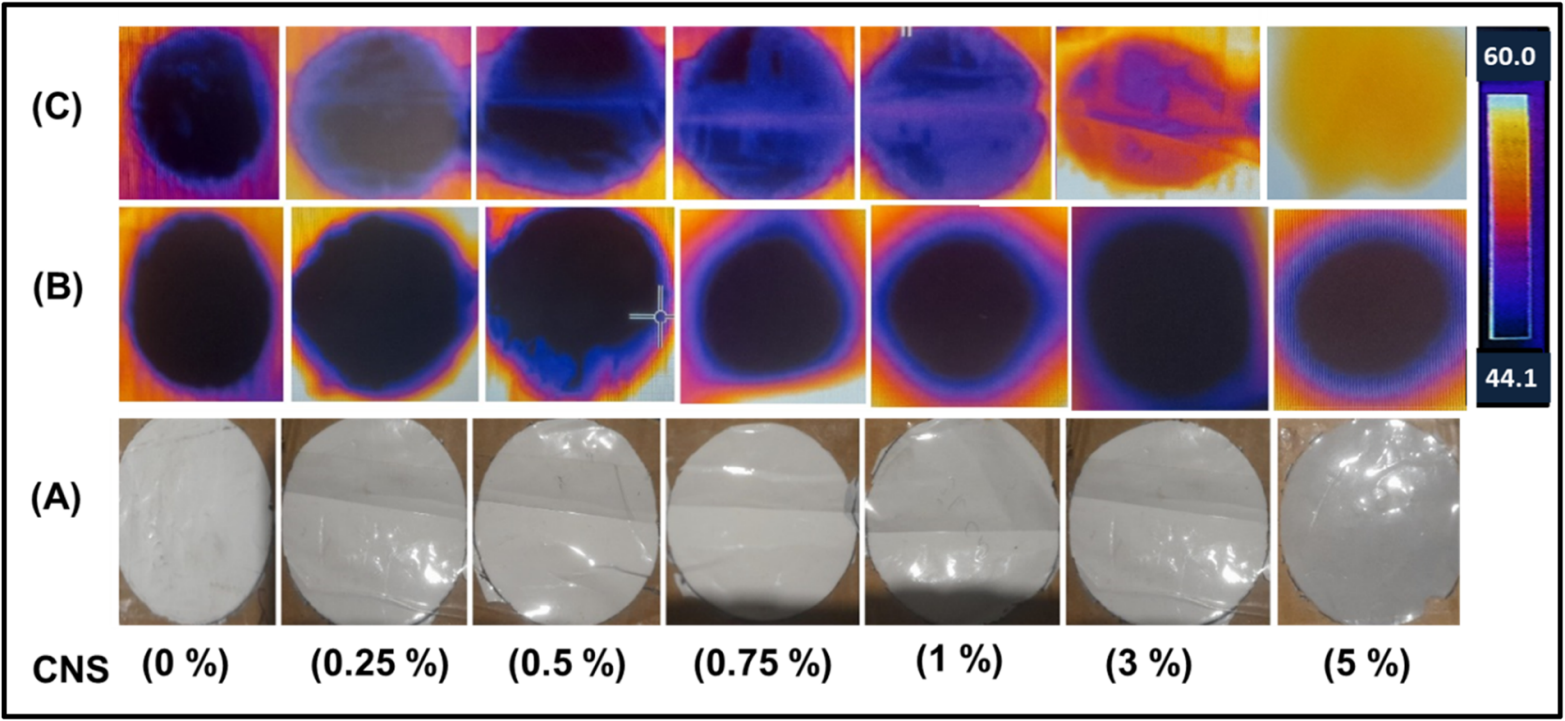
Photos of the membranes at different concentrations of CNS, (A) photos from an ordinary camera, (B) IR-thermal camera images of the membranes after 1 min. of exposure to sunlight, and (C) IR-thermal camera images of the membranes after 25 min. of exposure to sunlight (note: IR thermal camera photos have been taken outdoor at under direct solar radiation at 1 pm, August, Egypt).

Fig. 5 shows the thermal IR-camera images monitored after 1 and 25 minutes of exposure to sunlight. From the figure, the effect of the concentration of nano-carbon can be clearly seen, as the neat PSF membrane did not exhibit any thermal absorption at short and long periods of exposure to the sunlight. On the other hand, the nanocomposite membranes exhibited the highest thermal spectrum, especially at high concentrations of CNS and after 25 minutes of exposure to the sunlight. Practically, this behavior proves that CNS altered the thermal activity of the composite membrane. The membrane containing a high concentration of CNS (5%) exhibited the greatest temperature of 58.7 °C and thermal glow, whereas the neat PSF membrane recorded a temperature of 48.4 °C and lowest glow.

This phenomenon may be attributed to the π → π* transitions occurring on carbon atoms and isolation vibrations of bonds. The relaxation energy of these transitions is dissipated through surrounding molecules and released as heat on the surfaces of membranes, causing a rise in temperature across a broad range of wavelengths in the solar spectrum.^5,33^ Moreover, the uniform dispersion of CNS within the PSF matrix plays a crucial role in ensuring consistent thermal activity across the membrane surface. The presence of CNS not only contributes to the overall heat absorption but also facilitates the rapid distribution of thermal energy throughout the membrane, preventing localized hotspots and promoting a uniform thermal profile.^13^ This characteristic is particularly advantageous for applications requiring stable and sustained thermal output, such as in solar-driven water purification or thermal management systems.^1^

**Fig. 6:**
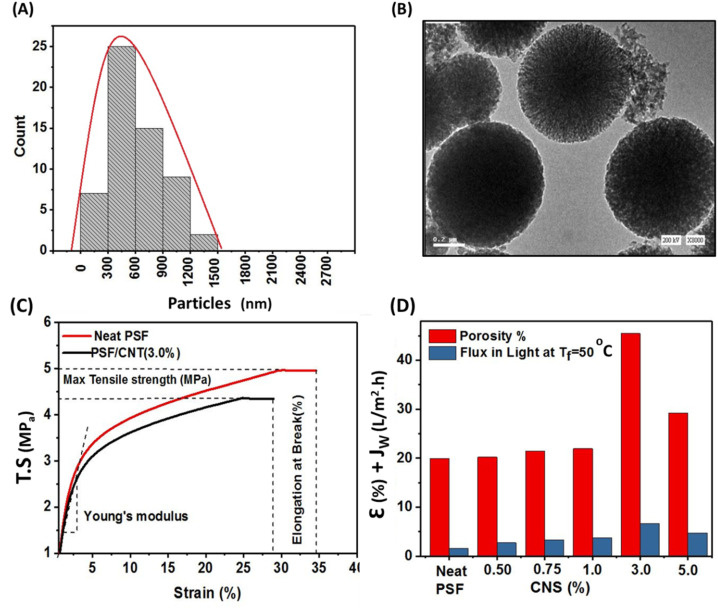
TEM particles size distribution of CNS (A), TEM image of CNS (B), DMA of neat PSF membrane compared to composite PSF/CNS (3.0%) membrane (C), porosity and highest flux in presence of light source at *T*_f_ = 50 °C of composite PSF/CNS compared to neat PSF membrane (D).

Fig. 6a and b Illustrates particles size distribution of carbon nanospheres which were determined and investigated by TEM images and originlab software where it was found that the maximum size was ranged from 300 nm to 780 nm as was depicted in Fig. 6a. While TEM images of carbon spheres was illustrated in Fig. 6b which demonstrated worm-like channels and homogeneously dispersed micropores can be seen in the carbon surface which confirm the results obtained from nitrogen adsorption–desorption isotherm.^22^

Fig. 6c depicts dynamic mechanical analysis (DMA) of neat PSF membrane compared to composite PSF/CNS (3%) as optimal membrane. Results showed that mechanical properties of the selected membranes there is a decrement in tensile strength (TS) from 5.0 ± 0.2 MPa to 4.6 ± 0.2 MPa, in elongation at break from 34.9 ± 0.3% to 27.5 ± 0.3% and in stress modulus (young's modulus) from 400 ± 32 MPa to 365 ± 29.5 MPa for neat PSF and modified PSF/CNS (3.0%) respectively. This decrement is due to the nanoparticles nature and its morphology and emphasized with Shahat *et al.* and Wu *et al.* results who suggested that increasing the concentration of nanomaterials could aggregate themselves and subsequently reduces the mechanical properties and causes the formation of macrovoids and microvoids in subsurface layer. These lead to the formation of more fragile membranes.^18^ Porosity (*ε*) calculated values of PSF and PSF/CNS composite membranes are shown in Fig. 6d which depicts that the highest value was in case of PSF/CNS (3%) is 45.46% compared to 20.41% in case of neat PSF and this may be attributed to change morphology effect of CNS which assisted to increase of water content for composite membranes due to creation of space voids and more porous subsurface layer with large pores at bottom like-channels which contribute to more efficient water flow through the membrane.^18^ Furthermore, the relationship between porosity and maximum permeate flux (PMD flux) under identical operational conditions is examined, highlighting the impact of porosity on membrane performance.

#### 3.2.1 Effect of CNS on water evaporation rate (ER)

**Fig. 7:**
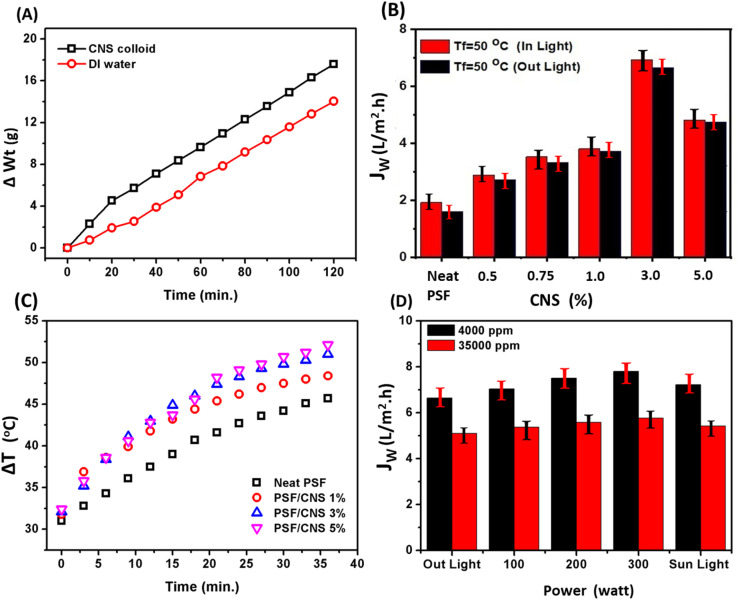
(A) Water evaporation rate curves of pure water and CNS suspension solution under LI = 1 sun in petri dish surface area = 0.0035 cm^2^, (B) effect of light on neat and composite PSF/CNS membrane surface at different concentrations of CNS (0%, 1%, 3%, 5%) at LI = 1 sun and *d* = 12 cm, (C) effect of CNS on PMD flux In and Out light at different *T*_f_ = 50 °C, (D) effect of various electrical light power sources and sunlight on PSF/CNS flux on synthesized NaCl water. Operation conditions: *T*_p_ = 20 °C, FFR = 1.4 L min^−1^, LI = 1 sun, feed water salinity = 4.0 g L^−1^.

3D porous carbon nanostructures are considered as promising materials for solar steam generation. For instance, a scalable 3D elastic cellular nitrogen-enriched structure enables high-efficiency *in situ* consequential photothermal vaporization using carbon sponge.^7,34^ In this section, our synthesized 3D micro-porous CNS showed broad-band with a varied wave length light of UV-spectroscopy range from 300 to 900 nm as illustrated in Fig. 3C, evaporation behavior of CNS is illustrated in Fig. 7a, asclearly an enhancement in rising of evaporation efficiency of 50 mL of DI water contains 0.3 mg of CNS that sonicated for 30 minutes and exposed to light source with incandescent electrical power 100 watts, it matches heat and brightness of one sun. At the beginning of the experiment, after 30 minutes, the evaporation rate of pure DI water and CNS colloid solution was 1.01 kg m^−2^ h^−1^, and 2.27 kg m^−2^ h^−1^, respectively. After 120 minutes, the value of ER of the pure DI water increased to 1.395 kg m^−2^ h^−1^ while that of CNS colloid solution decreased to 1.747 kg m^−2^ h^−1^. These results distinctly demonstrate the effect of CNS as a photothermal agent in water heating; however slightly decrease in ER with time may be due to increase CNS concentration with time which restricts evaporation process according to saline water physical roles.^20^

Fig. 7b compares the change in the surface temperature rates of PSF composite PSF/CNS membranes at a different content of CNS. It was found that increases in CNS content in the membrane matrix led to a significant increase in the surface's temperature of membranes with different rates. The recorded temperature after 35 minutes of exposure to the sunlight were 45.7, 48.4, 51.0, and 52.1 °C, respectively of the neat PSF and composite PSF/CNS composite membranes at different CNS concentrations of 1.0, 3.0, 5.0%, respectively. The enhancements result from the PSF skeleton and CNS's conjugated bonds C=C and S=O vibrations, which tightly held energy levels and absorbed the full spectrum of light through loosely π electrons. The absorbed light caused the π electrons to shift to the π* transition level, and then they released energy as heat through the membrane surface to return to their ground state.^6,29^ The liquid entry pressure (LEP) values of PSF and PSF/CNS composite membranes are shown in (ESI, Fig. S2†). LEP is a critical parameter in characterizing MD membranes as it represents the pressure difference needed for liquid penetration. The highest possible membrane efficiency depends on ensuring that the membrane pores remain free from liquid. From the figure, it is noticed that the LEP increased from 1.8 bar for pure PSF membrane to 5.4 bar for the modified membrane with 5% CNS and this accept with contact angles measurements which revealed increasing with CNS addition increase in prepared membranes as illustrate in (ESI, Fig. S3 and S4†) which show enhancements of contact angle from 78 ± 5° to 94 ± 5° for neat and modified with 5% CNS. This can be illustrated on the basis of that the addition of CNS inside the PSF acting as filler resisting the liquid water transport through the membrane pores as well as its hydrophobic nature (*i.e.* hydrophobic narrow pores). All these results practically confirm the significant improvement in the physical properties of the prepared membranes surface, particularly the enhancement of hydrophobicity, as demonstrated by contact angle measurements, in line with the scientific literature.^21,35^

### 3.3 Investigating the photothermal efficiency of the membranes

Fig. 7c depicts the effect of CNS concentration on vapor flux behavior of composite PSF/CNS membranes, which was examined at constant operation condition (*T*_f_ = 50 °C, *T*_p_ = 20 °C, FFR = 1.4 L min^−1^, feed water salinity = 4.0 g L^−1^ and LI ≈ 1 sun) in presence and absence of light. The composite membrane with a concentration of 3.0% of CNS exhibited the highest and optimal results, whereas it gave 6.93 L m^−2^ h^−1^ in light compared to 1.92 L m^−2^ h^−1^ for neat PSF. It was found that all PSF and composite PSF/CNS membranes have relatively higher water flux in the presence of light. These results are because of CNS microporous act as nanofillers, pores forms as well as photothermal conversion agents in light due to its very narrow band gap that assist in absorbance of sunlight in visible spectrum as a black body in varied wavelengths.^36^ Furthermore, they exhibit a significant capacity for converting solar energy into thermal energy, while maintaining a high level of stability. Conjugated systems of PSF can also exist, greatly improving the absorption of light across a large range of wavelengths and angles.^37^ All of these factors contribute to the enhancement of MD flux in a favorable direction. An ideal membrane for MD should possess a high liquid entry pressure (LEP), high hydrophobicity, low pore tortuosity, high void volume fraction (porosity), a narrow pore size distribution, low thermal conductivity, antifouling properties (good scaling resistance), and overall durability for long-term operation.^29^ The exposure of the composite PSF/CNS membrane to a light source, where CNS works as a photothermal agent, could reduce the temperature polarization on the membrane surface and hence increase the vapor pressure (*i.e.*, flux increases).

#### 3.3.1 Photonic energy effect on PMD flux of PSF/CNS membrane

Quantum theory states that the fundamental unit of optical power is the watt (W), defined as one joule (J) of energy per second. Planck's eqn (4) describes the energy carried by each photon.4
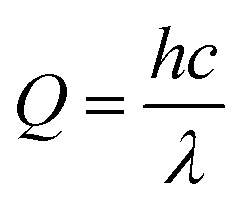
where *Q* is the photon energy, *h* is Planck's constant (6.623 × 10^−34^ J S^−1^), *c* is the speed of light (2.998 × 10^8^ m s^−1^), and *λ* is the wavelength of radiation in meters.

Increasing the electrical power results in more photons being emitted per unit of time, which leads to a greater intensity of light.^38^ This increase in photonic energy causes more electrons to excite and more π bonds to vibrate. Consequently, the lattice heat of the membrane surface increases.^37^ As a result, the temperature polarization gradient on the membrane surface decreases and the temperature difference between the hot and cold sides of the membrane becomes more pronounced. This reinforces the flux, which is illustrated in Fig. 7d. When the PMD system was exposed to various light intensities, including sunlight, the flux clearly increased with increasing light intensity. The highest flux of 7.81 L m^−2^ h^−1^ was achieved with a 300 watt electric power and light source (approximately 2.82 sun), compared to 6.648 L m^−2^ h^−1^ with no light source, under the same operational conditions (*T*_f_ = 50 °C, *T*_p_ = 20 °C, feed water salinity = 4.0 g L^−1^, and FFR = 1.4 L min^−1^). Furthermore, when seawater was used as the feed water, the maximum flux under the same conditions was 5.77 L m^−2^ h^−1^ at an intensity of approximately 2.82 sun, while the minimum flux was 5.1 L m^−2^ h^−1^ with no light, due to the effect of salinity.^34^

#### 3.3.2 Effect of feed temperature on PSF/CNS membrane flux

**Fig. 8:**
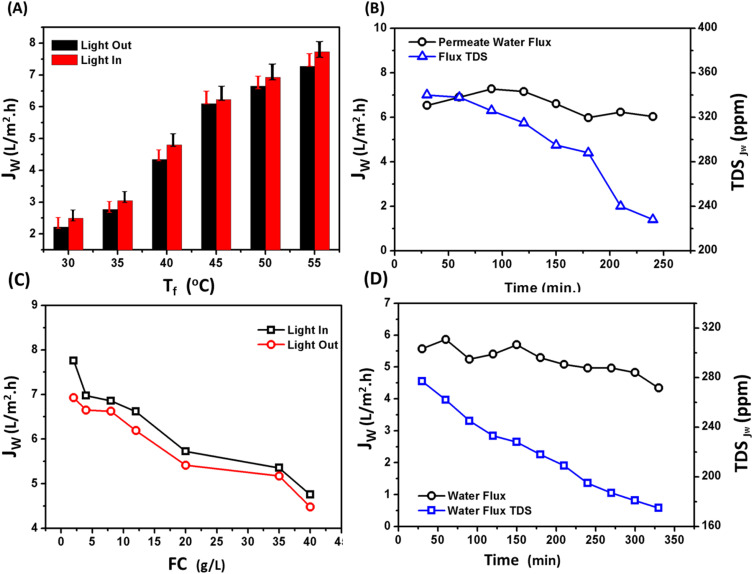
(A) Effect of feed temperature on the water flux of composite PSF/CNS membrane in presence of light source with an intensity of 1 sun. (B) Effect of feed water salinity of NaCl at constant *T*_f_ = 50 °C. (C) Durability of the composite PSF/CNS. (D) Desalination efficiency of a real sample of sea water collected from Suez Gulf (TDS = 38 g L^−1^) using the composite PSF/CNS membrane. Operation conditions: *T*_p_ = 20 °C, feed water salinity = 4.0 g L^−1^ and FFR = 1.4 L min^−1^.

The temperature of the feed has a significant impact on PSF/CNS membrane flux behavior. Fig. 8a shows that the flux of permeates increased as the temperature difference between the feed and permeate increased. When the temperature difference increased from 15 °C to 35 °C, the flux increased from 2.21 and 2.49 L m^−2^ h^−1^ to 7.28 and 7.73 L m^−2^ h^−1^. This was observed both in the absence and presence of light for *T*_f_ = 35 °C and 55 °C respectively, with a fixed *T*_p_ = 20 °C, FFR = 1.4 L min^−1^, feed water salinity = 4.0 g L^−1^, and a light source intensity of about 1 sun. This phenomenon may be attributed to the increased production of water vapor at the interface of the membrane, which facilitates the movement of water vapor *via* the pores because of a difference in vapor pressure.^33^

Nevertheless, increasing the temperature of the feed solution leads to an increase in the temperature gradient, which, in turn, amplifies the difference in vapor pressure that serves as the driving force for mass transfer. Consequently, it raises the vapor flux that permeates through the PMD system. Under the presence of a light source with an intensity of approximately 1 sun, a noticeable and distinct change occurs. In the absence of light, there is no active support for light. This phenomenon can be explained by the localized heat on the surface of the membrane, which occurs due to the conversion of light energy from CNS excitation relaxation electrons. This process also involves minor vibrations in the polymer chains. As a result, the temperature polarization on the feed side decreases.

#### 3.3.3 Effect of feed concentration on the water flux of PSF/CNS MD membrane

The mass transfer of water plays a crucial role in membrane distillation flux, which is affected by salinity of the feed water. It is basically known that increasing the feed water salinity leads to a higher boiling point of water, leading to a decrease in vapor pressure.^20^ The following equation illustrates the relation between water vapor temperature and water temperature:

5 *T*_v_ = *T*_b_ − BPE

where *T*_v_ and *T*_b_ are the temperature of evaporation and water boiling point temperatures, respectively.^34^Fig. 8b shows the effect of feed water salinity on the water vapor flux in presence and absence of the light source, generally the figure depicts that as the feed salinity increases from 2 g L^−1^ to 40 g L^−1^ the water flux gradually decreased from 7.756 and 6.924 L m^−2^ h^−1^ to 4.656 and 4.48 L m^−2^ h^−1^ in presence and absence of light, respectively. These results can be explained on the basis of the reduction of feed water vapor pressure, which reduce the rate of transmission of the water mass through membrane pores.

Fig. 8c demonstrates that the water flux of the composite PSF/CNS membrane remains relatively constant for duration of three hours. It is crucial to note that the membrane had been utilized for more than 40 hours prior to conducting this experiment, and the flux remained consistently stable at 6.93 L m^−2^ h^−1^. Consequently, the presence of nanomaterials and conducting the experiment at low temperature effectively prevented membrane wetting and fouling. Furthermore, the figure clearly demonstrates a consistent drop in the total dissolved solids (TDS) of the permeate flux when the salinity of the distillate side decreases from 340 ppm to 228 ppm. This unequivocally validates the membrane's strong salt rejection capability, which is around 99.99%.

#### 3.3.4 Scaling effect of Suez Gulf water on PSF/CNS membrane

Mineral scaling is a significant problem for membrane desalination. It limits the performance of the process and poses a major challenge for desalination technologies.^18^ When feed waters become concentrated in desalination, solute concentrations go beyond the solubility of sparingly soluble solids. As a result, mineral scales form on the membrane surface.^25^ The presence of minerals on the membrane surface reduces water flux and membrane lifespan in desalination systems. This affects the cost and energy efficiency of the systems.^39–41^ To address this issue, a new modified composite PSF/CNS membrane with a concentration of 3.0% was tested using the PMD unit. The feed water used was from the Suez Gulf, which contains various salts, pollutants from ships waste, and oils from surrounding oil companies. Tap water with a TDS of 277 ppm was used in the permeate tank. The flow rate was 1.4 L min^−1^ and the intensity of light (LI) was approximately 1 sun. Fig. 8d illustrates slightly effect of membrane flux which reduced from 5.36 L m^−2^ h^−1^ (average of first 60 minutes) to (4.73 L m^−2^ h^−1^ average of last 60 minutes) after 320 minutes as operation time interval also sloped decline showed in TDS curve of permeate from 277 ppm to 175 ppm due to mixing of vapor zero salts with tap water of permeate tank.

Comparison of PSF/CNS photothermal membrane with previous works; comparative Table S1 has been documented in the ESI† between our work and previous composite polysulfone membranes in membrane distillation works.

## 4. Conclusion

Hydrothermal synthesis of photothermal microporous carbon nanospheres (CNS) at 200 °C, followed by pyrolysis at 700 °C, resulted in particles characterized using various analytical techniques. CNS inclusion in polysulfone (PSF) membranes improved photothermal efficiency, enhancing membrane distillation flux. Wet phase inversion method facilitated preparation of neat PSF and PSF/CNS membranes, confirming enhanced photothermal characteristics through IR-camera images. CNS presence increased evaporation rate in DI water. Overall, hydrothermal CNS exhibited notable photothermal properties, enhancing polysulfone MD membrane flux through light absorption and conversion to heat. The optimal CNS concentration in PSF was found to be 3%, leading to improved morphology and new photothermal attributes.

## Data availability

The data supporting this article have been included as part of the ESI.†

## Conflicts of interest

There are no conflicts to declare.

## Supplementary Material

RA-014-D4RA05629A-s001
