# Supplementary material for: Photothermal effectiveness of microporous carbon nanospheres incorporated with polysulfone in direct contact membrane distillation
Source: RSC Adv. 2024 Sep 27;14(42):30912–23. doi: 10.1039/d4ra05629a (PMC11427996; doi:10.1039/d4ra05629a)
Supplement: RA-014-D4RA05629A-s001 [file RA-014-D4RA05629A-s001.pdf]

Supplementary Information (SI) for RSC Advances.
This journal is © The Royal Society of Chemistry 2024

# Photothermal effectiveness of microporous carbon nanospheres incorporated with polysulfone in direct contact membrane distillation

**Moataz Morad[1], Mohamed S. Fahmi[2], Abdu Subaihi[3], Mohammed T. Alotaibi[4], Ahmed Shahat[2], Mohamed E. A. Ali[5*]**

[1] Chemistry Department, Faculty of Sciences, Umm Al-Qura University, 21955 Makkah, Saudi Arabia.
[2] Chemistry Department, Faculty of Science, Suez University, P.O. Box: 43221, Suez, Egypt.
[3] Department of Chemistry, University College in Al-Qunfudhah, Umm Al-Qura University, Saudi Arabia.
[4] Department of Chemistry, Turabah University College, Taif University, P.O. Box 11099, Taif 21944, Saudi Arabia.
[5] Egypt Desalination Research Center of Excellence, Hydrogeochemistry Department, Desert Research Center, Cairo 11753, Egypt.

Corresponding author; m7983ali@drc.gov.eg

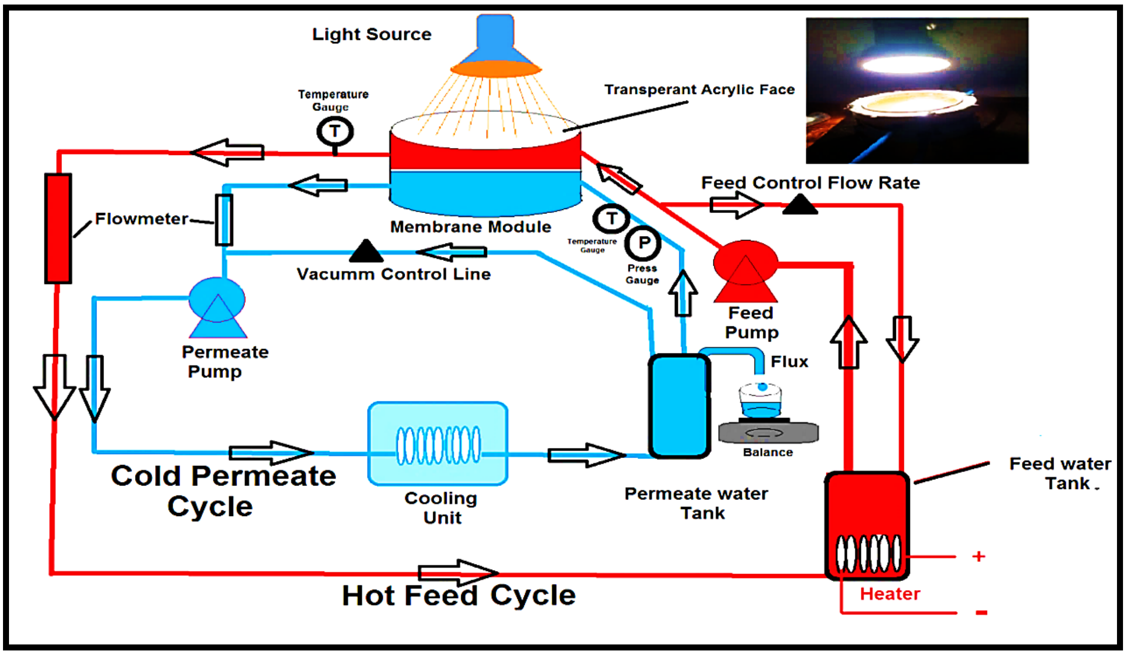


**Figure S1**. Schematic representation of the lab Scale Photothermal Direct Contact Membrane Distillation System

## 1. Synthesis of Carbon Nano-Spheres (CNS)

CNS particles were prepared by applying the green chemistry approach [1]. To summarize, 4.0 g of D-glucose (carbon precursor) was dissolved in 60 ml of deionized water (DI). Next, it was enclosed and heated in an electric furnace at 200 °C for 8 hours, followed by cooling to ambient temperature. Next, the resulting dark product was collected through filtration, washed with DI water and ethanol multiple times, and subsequently dried at 80 °C overnight. The produced CNS was subjected to calcination in an electric tube furnace at 700°C for 4 h under a nitrogen gas atmosphere.

## 1.2.

## Characterization of CNS particles and membranes

Field emission scanning electron microscopy model (Zeiss FESEM Ultra 60) operated with 5 kV to investigate the surface morphology of the membranes and cross sections. Micromeritics ASAP-2020 was used to perform nitrogen adsorption - desorption isotherms at -196 °C, where the samples were degassed first for 4 h at 100 °C to remove any humidity or contaminants. To evaluate the values of surface area,

the Brunauer Emmett-Teller (BET) method was employed. In addition, the Pore size distribution of the produced CNS was calculated from the nitrogen isotherm adsorption branch via the Barrett-Joyner-Halenda (BJH) approach. The surface area and volume of micropore were analyzed by applying the t-plot method. Raman measurement was conducted at a 532 nm laser beam wavelength Raman microscope (Pro Raman-L Analyzer). The functional groups of CNS and membranes were investigated using Fourier transform infrared spectroscopy (FT-IR); the measurements were conducted using a BRUKER Vertex 70 FT-IR spectrometer. Infrared spectroscopy measurements have been completed over a wide range of frequencies, allowing measuring the evolution of both intra-molecular and inter-molecular vibrational modes. The membranes were scanned at a wavenumber of 400–4000 $cm^{-1}$, in order to investigate changes in the functional groups of the pristine and composite membranes and High-resolution transmission electron microscope (HRTEM) images were taken by JEOL JEM-2100 Electron Microscope. Absorption wavelenghts range for CNS as a photothermal agent was determined by the compact UV-spectrophotometer model UV-2600i in wavelength was randed form 240 to 900 nm at different suspension concentration of CNS 0.03%, 0.015% and 0.0075%. The light-to-heat conversion, and the effects of structural features in the development of broadband light-absorbing nanomaterials[2, 3] is the essential requirement and prerequisite which can be defined as:

$$A = \frac{\int_{\lambda 1}^{\lambda 2} (1 - R - T) S_{solar}\, d\lambda}{\int_{\lambda 1}^{\lambda 2} S_{solar}\, d\lambda} \quad \text{.............................. (1)}$$

where A, R, and T are the standards for absorbance, reflectance, and transmittance, respectively, S solar is the wavelength-dependent, $\lambda_1$ and $\lambda_2$ are the integration beginning and end wavelengths, respectively [4-6]. Water contact angle of CNS was measured at room temperature using sessile drop technique on the drop shape analyzer where CNS pressed sheet were wetted with 3 μL of distilled water via thin needle to form a tiny droplet onto the sample surface add to that different prepared membranes were measured with same steps . Evaporation rate was evaluated and calculated from equation(2) for DI water compared to CNS suspension water by using light source 100

wat (about 1 sun ) at distance 12 cm from Pateri dish with surface area(0.00385 $m^2$) contains of 50 ml from investigated liquid.

$$E.R = \Delta W / ( A \times \Delta t ) \quad \ldots\ldots\ldots\ldots\ldots\ldots \quad (2)$$

Where ($\Delta W$) signed to weight change per kg, (A) refers to surface area per $m^2$ and ($\Delta t$) evaporating rate time per hour.

The dynamic mechanical analysis was performed using a Dynamic Mechanical Analyzer (DMA) (Model DMA Q800 V21.1 Build 51 - Controlled Force) where the samples, cut into strips measuring 40 mm in length and 10 mm in width, were subjected to mechanical testing. The measurement of cross-sctional thickness of the membranes, a precise thickness gauge was employed. The mechanical properties were quantified as follows [7]:

$$Tensile\ Strength\ (T.S) = \frac{F}{Ao} \quad \text{(MPa)} \ldots\ldots\ldots\ldots (3)$$

Where F is the force applied (N) and $A_o$ is the original cross-sectional area of the membrane sample ($mm^2$).

$$Elongation\ rate = \frac{\triangle L}{Lo} \quad (\%) \ldots\ldots\ldots\ldots\ldots\ldots (4)$$

Where $\Delta L$ is the change in length (mm) and L0 is the original length of the membrane sample (mm).

$$\text{Young s Modulus (E)} = \frac{tensile\ strength}{tensile\ strain} \quad \text{(MPa)} \ldots\ldots\ldots\ldots (5)$$

Liquid Entry Pressure (LEP), it was measured for all the membranes by using a dead end cell (HP4750 Stirred Cell) system, which was connected to a nitrogen gas cylinder as inert gas to apply the required pressures using distilled water as a liquid [8, 9]

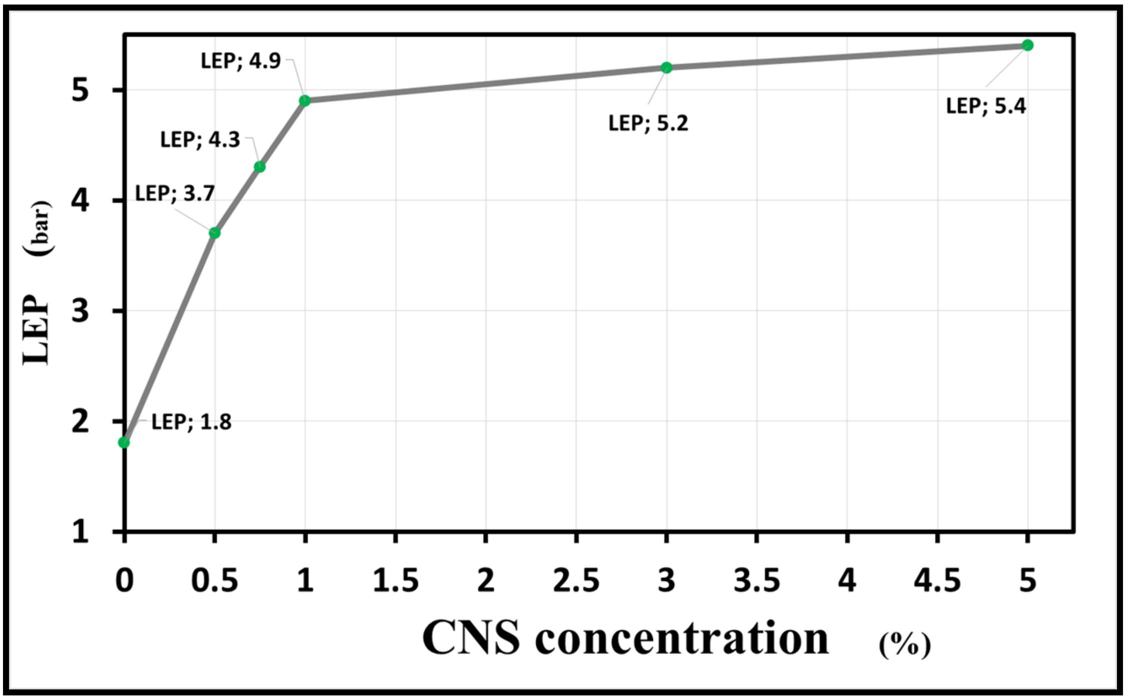


**Figure S2.** LEPs of different PSF/CNS composite membranes at room temperature by dead end Nano filtration cell, $N_2$ cylinder as pressure source and DI water as a liqu

**Evaluation of Porosity of prepared membranes compared to flux values**

The total Porosity (ε) (%) of different prepared membranes was determined after immersion in DI water for 24 hours at room temperature and weighted after swiping excess of water by filter paper ($W_w$) (g) and then dry in 50 °C for 4 hours and take dry weight ($W_d$) (g) and calculated by equation (3):

$$\varepsilon = \frac{\frac{Ww - Wd}{\rho 1}}{\frac{Ww - Wd}{\rho 1} + \frac{Wd}{\rho 2}} x100 \quad (\%)\ldots\ldots\ldots\ldots\ldots\ldots\ldots\ldots\ldots\ldots\ldots\ldots (6)$$

Where $\rho_1$ is DI water density (0.998 g/cm$^3$) and $\rho_2$ is density of polysulfone (1.24 g/cm$^3$) [10].

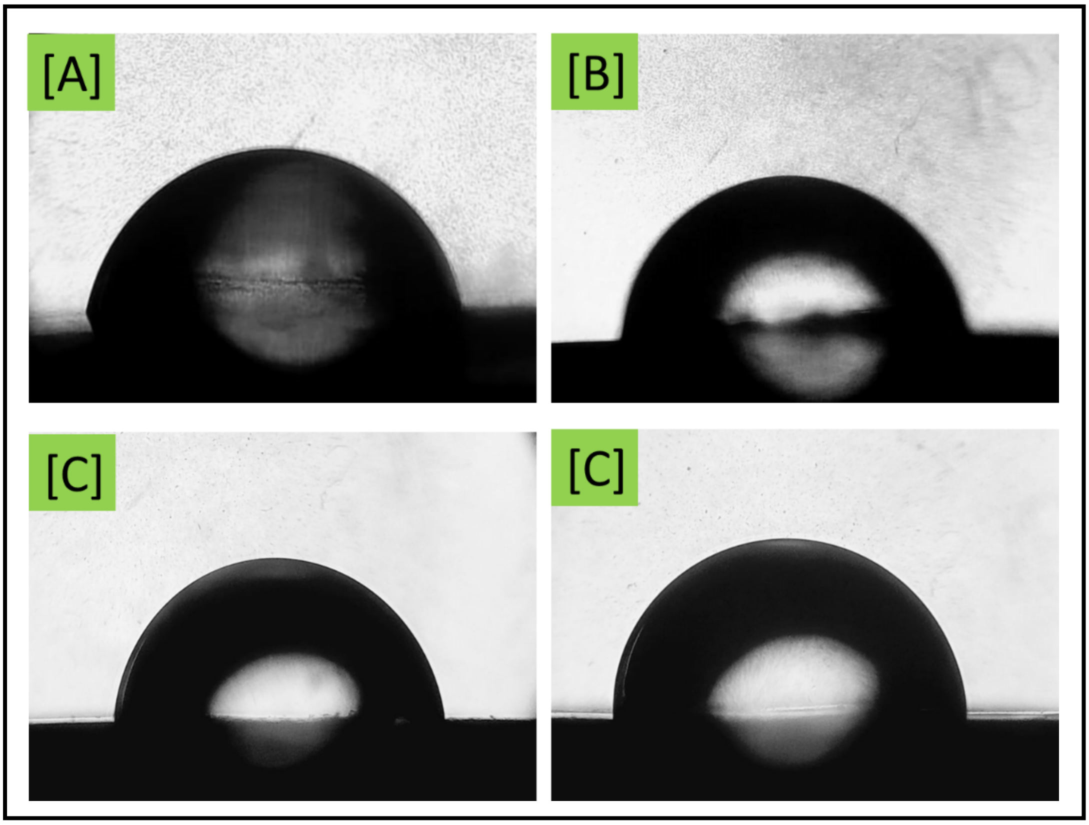


**Fig. S3:** Illustrates images of different PSF membranes contact angles measurements according to CNS percent A)Neat PSF B)CNS (0.5%) C) CNS (3%) D) CNS (5%)

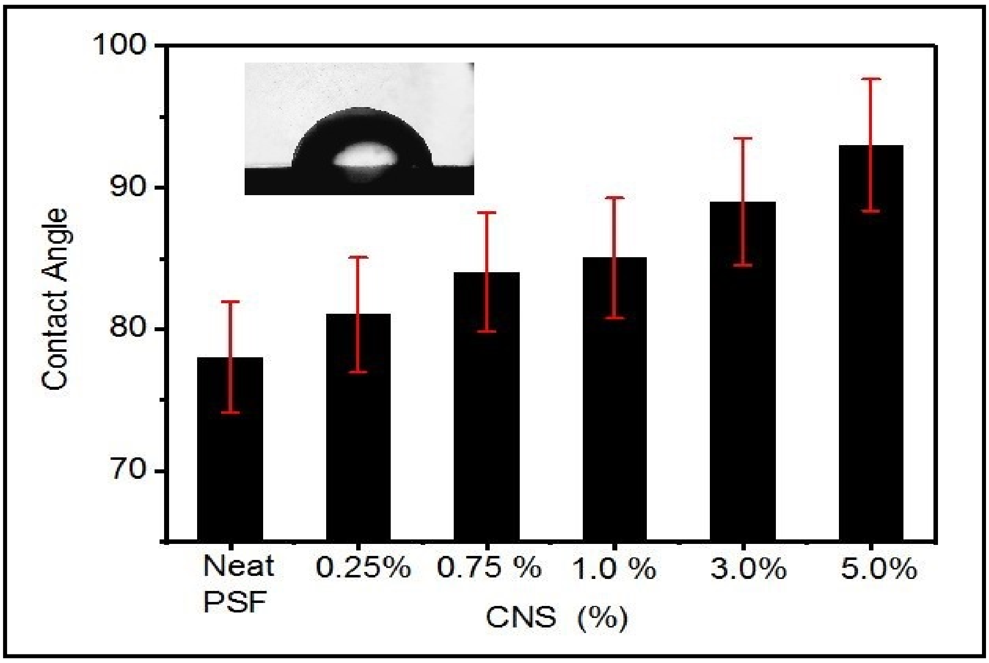


**Fig. S4:** Illustrates contact angle measurements of pristine ad composite PSF/CNS membranes at room temperature.

**Table S1. A comparing of photothermal composite PSF/CNTs membranes with some composite polysulfone previous works.**

| **Composite Membrane** | **Object** | **MD configuration** | **Flux Value L/m$^2$.h** | **Salts rejection** | **Operation conditions** | **Ref.** |
|---|---|---|---|---|---|---|
| PSF/CuO | Enhancement of photothermal properties | PMD | 9.87 | 99.6 % | $T_F$=60 °C $T_P$=20 °C FC=123,14 mg/L | [11] |
| PSF/PTFE NPs | The transition from hydrophobic to superhydrophobic | DCMD | 39.5 | 99.99 % | $T_F$=60 °C $T_P$=20 °C FC=30 g/L NaCl | [12] |
| GO-PSF | Enhancement morphology | DCMD | 20.8 | 99.85 % | $T_F$=90 °C $T_P$=20 °C FC=20.8 g/L NaCl | [13] |
| PS/PEG/$Al_2O_3$<br>PS-PEG/CuO<br>PS-PEG/SWCNTs<br>PS/PEG/MWCNTs | Enhancement Morphology | EDCMD | (19.92)<br>(18.92)<br>(20.91)<br>(18.20) | 99.99 % | $T_F$=60 °C $T_P$=20 °C FC=20 g/L NaCl | [14] |
| PSF/CNS | Enhancement Of photothermal properties and morphology | PDCMD | 7.756 | 99.99% | $T_F$=50 °C $T_P$=20±3 °C FC= 2.0 g/L | Our work |
| PSF/MWCNTs<br>PSF/$SiO_2$<br>PSF/ZnO<br>PSF/$TiO_2$ | Enhancement morphology | VEDCMD | 41.58<br>34.42<br>38.84<br>35.6 | 99.99 % | $T_F$=60 °C $T_P$=20 °C FC=10 g/L NaCl | [15] |

## Abbreviation

PMD : Photothermal Membrane Distillation

DCMD : Direct Contact Membrane Distillation

EDCMD : Direct Contact Membrane Distillation

PDCMD : Photothermal Direct Contact Membrane Distillation

VEDCMD: Vacuum Enhanced Direct Contact Membrane Distillation

Tf : Feed temperature

Tp : Permeate temperature

FC : Feed concentration

## Referances

[1] S.K. Mohamed, M. Abuelhamd, N.K. Allam, A. Shahat, M. Ramadan, H.M. Hassan, Eco-friendly facile synthesis of glucose–derived microporous carbon spheres electrodes with enhanced performance for water capacitive deionization, Desalination, 477 (2020) 114278.

[2] M. Xiong, X. Shan, C. Liu, L. Yang, M. Meng, Y. Di, Z. Gan, Broadband photodetectors based on enhanced photothermal effect of polymer encapsulated graphene film, Applied Surface Science Advances, 3 (2021) 100050.

[3] P. Cheng, D. Wang, P. Schaaf, A review on photothermal conversion of solar energy with nanomaterials and nanostructures: From fundamentals to applications, Advanced Sustainable Systems, 6 (2022) 2200115.

[4] M. Gao, L. Zhu, C.K. Peh, G.W. Ho, Solar absorber material and system designs for photothermal water vaporization towards clean water and energy production, Energy & Environmental Science, 12 (2019) 841-864.

[5] L. Zhu, M. Gao, C.K.N. Peh, G.W. Ho, Solar-driven photothermal nanostructured materials designs and prerequisites for evaporation and catalysis applications, Materials Horizons, 5 (2018) 323-343.

[6] R. Xu, H. Zhao, H. Jin, Z. Wang, Z. Zhang, S. Xu, Z. Zeng, S. Wang, Y. Lei, Scalable fabrication of geometry-tunable self-aligned superlattice photonic crystals for spectrum-programmable light trapping, Nano Energy, 58 (2019) 543-551.

[7] Rugbani, A. (2009). Investigating the influence of fabrication parameters on the diameter and mechanical properties of polysulfone ultrafiltration hollow-fibre membranes (Doctoral dissertation, Stellenbosch: University of Stellenbosch).

[8] G. Rácz, S. Kerker, Z. Kovács, G. Vatai, M. Ebrahimi, P. Czermak, Theoretical and experimental approaches of liquid entry pressure determination in membrane distillation processes, Periodica Polytechnica: Chemical Engineering, 58 (2014) 81-91.

[9] M. Khayet, C. Feng, K. Khulbe, T. Matsuura, Preparation and characterization of polyvinylidene fluoride hollow fiber membranes for ultrafiltration, polymer, 43 (2002) 3879-3890.

[10] Velu, S., Muruganandam, L., & Arthanareeswaran, G. (2015). Preparation and performance studies on polyethersulfone ultrafiltration membranes modified with gelatin for treatment of tannery and distillery wastewater. Brazilian Journal of Chemical Engineering, 32, 179-189.

[11] Kamiński, W., B. Opara, and M. Tylman. "Membrane distillation using solar energy–membrane modification." Innovations 7, no. 3 (2019): 115-117.
[12] M.S. Fahmey, A.-H.M. El-Aassar, M.M. Abo-Elfadel, A.S. Orabi, R. Das, Comparative performance evaluations of nanomaterials mixed polysulfone: A scale-up approach through vacuum enhanced direct contact membrane distillation for water desalination, Desalination, 451 (2019) 111-116.
[13] Camacho, Lucy M., Trent A. Pinion, and Samuel O. Olatunji. "Behavior of mixed-matrix graphene oxide–polysulfone membranes in the process of direct contact membrane distillation." Separation and Purification Technology 240 (2020): 116645.
[14] Shahlol, Osamah MA, Heba Isawi, Mohamed G. El-Malky, and Abd El-Hameed M. Al-Aassar. "Performance evaluation of the different nano-enhanced polysulfone membranes via membrane distillation for produced water desalination in Sert Basin-Libya." Arabian Journal of Chemistry 13, no. 4 (2020): 5118-5136.
[15]Khayet, Mohamed, and Rong Wang. "Mixed matrix polytetrafluoroethylene/polysulfone electrospun nanofibrous membranes for water desalination by membrane distillation." ACS applied materials & interfaces 10, no. 28 (2018): 24275-24287.
